# Artificial intelligence in cancer genomics: refining diagnosis of hereditary breast and ovarian cancer syndromes

**DOI:** 10.1097/MS9.0000000000004340

**Published:** 2025-11-18

**Authors:** Muhammad Khizar, Muhammad Zaib, Meerab Babar, Hasiba Karimi, Hasibullah Aminpoor

**Affiliations:** a Faculty of Medicine, Georgian American University, Manifest Medical Research Co., Tbilisi, Georgia; b Faculty of Medicine, Rawalpindi Medical University, Rawalpindi, Pakistan; c Faculty of Medicine, Bezmialem Vakif University, Istanbul, Turkey; d Faculty of Medicine, Kabul University of Medical Sciences “Abu Ali Ibn Sina”, Kabul, Afghanistan

**Keywords:** artificial intelligence, genomics, precision oncology, soft tissue sarcoma

## Abstract

The integration of artificial intelligence (AI) into soft tissue sarcoma (STS) diagnostics represents a significant leap toward precision oncology. AI-driven genomic tools, especially in the detection of fusion genes, enhance diagnostic sensitivity, speed, and accuracy, facilitating improved patient stratification and personalized therapy. The convergence of AI with multi-omic data – including transcriptomic, epigenomic, and imaging datasets – offers a comprehensive view of sarcoma biology and supports earlier detection and optimized treatment selection. However, effective implementation requires international collaboration, standardized reporting frameworks, and continuous clinician education. AI holds transformative potential to advance the precision, efficiency, and clinical impact of STS management.

*Dear Editor*,

Hereditary breast and ovarian cancer (HBOC) syndromes, primarily due to germline BRCA1/2 mutations, significantly elevate lifetime cancer risk. BRCA1/2 mutations account for 10–20% of all ovarian cancers, with mutation carriers facing up to a 44% lifetime risk^[1]^. Early detection is critical, enabling preventive strategies and surveillance. In high-income countries like the UK, universal BReast CAncer (BRCA) testing for ovarian cancer is now considered cost-effective^[1]^. However, access remains limited in low- and middle-income countries. In India, population-wide BRCA screening could prevent nearly 97 000 ovarian cancer cases and 78 000 deaths^[1]^, underscoring the global need for scalable, cost-effective HBOC diagnostics.

Artificial intelligence (AI) offers promising solutions across the HBOC care continuum. AI-powered chatbots, for instance, are being used to pre-screen patients based on family history. A US study using the chatbot “Gia” found that trust, cost, and physician communication influenced patient engagement^[2]^. Nonetheless, such tools can help extend genetic screening to underserved and rural populations. Similar automated prescreening tools are being deployed in the UK and Australia and hold promise for low-resource settings.

AI also enhances variant interpretation. Next-generation sequencing often detects rare variants of uncertain significance, complicating clinical decision making. Machine learning (ML) tools such as MARGINAL apply ACMG-AMP criteria to classify BRCA1/2 variants with up to 98% accuracy^[3]^. BRCA-specific ML classifiers also outperform generic models in pathogenicity prediction^[4]^, improving consistency in genetic test interpretation. As gene panels expand to include PALB2, RAD51, and TP53, ML algorithms incorporating population data and conservation scores will be essential, especially if trained on diverse, multiethnic cohorts.

AI applications are also advancing tumor genomics. A notable example is DeepHRD, a convolutional neural network that predicts homologous recombination deficiency (HRD) from H&E pathology slides^[5]^. Trained on over 1400 breast and ovarian tumors, DeepHRD achieved an AUC of 0.81 and identified 1.8–3.1 times more HRD-positive cases than standard assays^[5]^. This approach can reveal candidates for targeted therapies like PARP inhibitors, directly from routine pathology.

In summary, AI is transforming HBOC diagnosis from improving risk screening to enhancing variant classification and detecting genomic signatures via histopathology. These technologies offer scalable, equitable solutions, particularly for underserved populations. However, their integration requires careful clinical validation, diverse data representation, ethical oversight, and clinician education. With global collaboration, AI can help close the gap in hereditary cancer detection and care. We confirm that no AI tools were utilized in the conduct or writing of this manuscript, in accordance with the TITAN checklist^[6]^.

## Data Availability

Not applicable.
